# Research on Damage Evolution Laws and Life Prediction of 12Cr1MoVG Heat-Resistant Steel Under Different Thermal Shock Cycles

**DOI:** 10.3390/ma19183849

**Published:** 2026-09-10

**Authors:** Yanmiao Qu, Shiyu Li, Weihui Xu, Xinwei Guo, Weishu Wang

**Affiliations:** 1 Henan Provincial Institute of Boiler and Pressure Vessel Inspection Technology and Science, Zhengzhou 450016, China; qumiao217@126.com; 2 College of Energy and Power Engineering, North China University of Water Resources and Electric Power, Zhengzhou 450045, China; 16603848224@126.com (S.L.); xuweihui@ncwu.edu.cn (W.X.); guoxinwei1991@126.com (X.G.)

**Keywords:** thermal shock, material fatigue damage, equivalent plastic strain, life prediction

## Abstract

When a thermal power unit operates under deep peak shaving and variable load conditions, the heat-resistant materials of the boiler’s heat exchange surfaces will suffer accelerated fatigue damage due to frequent thermal shocks. To grasp the evolution law of thermal shock damage in high-temperature heat-resistant steel for the key equipment of thermal power units, based on the 12Cr1MoVG heat-resistant steel, a plastic strain simulation analysis was conducted. Through numerical simulation, the coupling relationship among thermal shock duration, thermal stress evolution, equivalent plastic strain (PEEQ) accumulation, damage penetration depth, and fatigue life was investigated. The results show that extending the duration of thermal shock will increase the thermal stress of the material, causing the failure depth to increase from 2.24 mm to 2.6 mm, and the accumulation rate of PEEQ at different depths of the material to accelerate, with the theoretical life decreasing from 1.82 × 10^5^ cycles to 1.72 × 10^5^ cycles. Extending the duration of low-temperature exposure will reduce the thermal stress of the material, causing the failure depth to decrease from 2.24 mm to 2.03 mm, and the accumulation rate of PEEQ at different depths of the material to slow down, with the theoretical life increasing from 1.81 × 10^5^ cycles to 1.94 × 10^5^ cycles. The research results can provide reference for fatigue damage and life assessment of the high-temperature and high-pressure materials used in key equipment of thermal power.

## 1. Introduction

The global energy sector is undergoing a rapid transition toward clean and low-carbon development. The increasing penetration of renewable energy has imposed new requirements on the flexibility and stability of power systems. As an important source of basic power supply and system regulation in China, coal-fired power generation is gradually shifting from conventional base-load operation toward more flexible peak-shaving and frequency-regulation services [1]. Consequently, deep peak-shaving operation and rapid load variations have become increasingly common operating conditions for thermal power units [2,3]. Frequent load fluctuations expose boiler heat-transfer surfaces to repeated temperature variations and thermal shocks, which may accelerate structural degradation and fatigue damage of heat-resistant materials and consequently increase the safety risks associated with long-term operation.

Previous studies have investigated thermal fatigue and failure mechanisms of high-temperature pressure-bearing components using numerical simulation, experimental testing, and failure analysis. Zhao et al. [4] investigated high-cycle thermal fatigue in primary-loop pressure-bearing pipelines and demonstrated that the frequency of temperature fluctuations significantly affects temperature attenuation near weld regions. Zhang et al. [5] reported that secondary stress induced by structural constraints, together with cyclic thermal stress, contributed to leakage failure of a low-pressure bypass drainage pipeline. Failure analyses of boiler superheater tubes and high-temperature drainage components have further demonstrated that cyclic thermal loading can promote crack initiation, crack propagation, and structural degradation [6,7]. Han et al. [8] developed a thermal-fatigue testing method for pressure-bearing pipelines and combined experiments with finite element analysis to evaluate crack-growth behavior under transient temperature variations.

Material degradation of heat-resistant steels under long-term high-temperature service has also received considerable attention. He et al. [9] investigated the failure behavior of heat-resistant steels under deep peak-shaving conditions and demonstrated the important influence of stress and high-temperature degradation on material failure. Wang et al. [10] analyzed a 12Cr1MoV superheater tube after 280,000 h of service and reported degradation of mechanical properties accompanied by creep voids and macroscopic cracking. Other investigations have shown that local overheating, corrosion-induced wall thinning, welded-region defects, and geometrical transition regions can also contribute to the degradation and failure of boiler tubes [11,12,13,14]. More specifically, Mohtadi-Bonab et al. [15] investigated long-term-serviced 12Cr1MoV superheater tubes and demonstrated the important roles of microstructural degradation and crack propagation in tube rupture. Shen et al. [16] experimentally characterized early creep degradation of 12Cr1MoVG steel at elevated temperature and related the observed microstructural evolution to progressive material degradation. Yu et al. [17] investigated cracking of a 12Cr1MoVG reheater outlet header under high-temperature and high-pressure service conditions. More recently, Man et al. [18] systematically investigated the high-temperature mechanical and creep behavior of 12Cr1MoVG steel and combined creep-life modeling with finite element analysis. Shen et al. [19] further characterized the microstructural and mechanical degradation of 12Cr1MoVG steel after thermal aging over a temperature range of 400–800 °C. These recent investigations demonstrate that temperature-dependent material degradation, creep, and microstructural evolution are important considerations in the assessment of 12Cr1MoVG components.

Although these studies provide important insights into the failure and degradation mechanisms of high-temperature pressure-bearing components, most previous investigations have focused on post-service failure analysis, crack initiation and propagation, corrosion degradation, or long-term creep behavior. The progressive damage evolution induced specifically by repeated thermal shock remains insufficiently quantified, particularly with respect to the coupled relationship among thermal stress evolution, equivalent plastic strain (PEEQ) accumulation, damage penetration depth, and fatigue-life degradation. Furthermore, the effects of individual thermal-cycle parameters, such as high-temperature thermal shock duration and low-temperature holding duration, on the through-thickness evolution of plastic strain and theoretical fatigue life have not been systematically clarified.

Therefore, the present study investigates the thermal shock-induced damage evolution of 12Cr1MoVG heat-resistant steel through thermo-mechanical finite element analysis. The effects of thermal shock duration and low-temperature holding duration on thermal stress evolution, PEEQ accumulation at different depths, damage penetration behavior, and theoretical fatigue life are systematically evaluated. The main contributions of this study are as follows: (1) the evolution characteristics of thermal stress and PEEQ accumulation under different thermal-cycle conditions are clarified; (2) the influence of thermal shock duration and low-temperature holding duration on damage penetration through the material thickness is quantitatively evaluated; and (3) the relationship between plastic strain accumulation and theoretical fatigue-life variation is analyzed, providing a numerical basis for understanding the influence of thermal-cycle parameters on the fatigue degradation of 12Cr1MoVG steel.

## 2. Model and Calculation Method

### 2.1. Mathematical and Physical Model

#### 2.1.1. Heat Conduction Equation

During the heating process, heat transfer within the material follows Fourier’s law of thermal conduction:

(1) q=−k∇T

where *q* is the heat flux density (W/m^2^), *k* is the thermal conductivity of the material (W/(m·K)), and ∇*T* is the temperature gradient (K).

For transient heat transfer processes, the governing equation can be expressed as:

(2) $$\frac{\partial T}{\partial t}=\alpha \nabla^{2}T$$

where $\alpha =k/(\rho c_{p})$ is the thermal diffusivity of the material, ρ is the material density (kg/m^3^), and c_p_ is the specific heat capacity (J/(kg·K)).

#### 2.1.2. Thermal Stress Equation

According to the principle of heat conduction, thermal stress is generated inside the material during heating.

The thermal stress can be described by:

(3) σ=EαΔT

where *σ* is the thermal stress generated during heating, (MPa), *E* is Young’s modulus of the material, MPa, *α* is the coefficient of thermal expansion, (°C^−1^), and Δ*T* is the temperature variation during heating, (°C).

This study focuses on the fatigue behavior of heat-transfer tube wall materials subjected to repeated thermal shocks. The thermal stresses generated in the tube wall mainly consist of radial thermal stress and circumferential thermal stress [20,21,22]. These stresses can be described by Equations (4) and (5):

(4) $$\sigma_{r}=\frac{C_{1}}{r^{2}}+C_{2}(1+\ln r)+\frac{\alpha E}{1-\upsilon }\cdot \frac{1}{r^{2}}\int rT(r)dr$$

(5) $$\sigma_{\theta }=-\frac{C_{1}}{r^{2}}+C_{2}(3+\ln r)+\frac{\alpha E}{1-\upsilon }\cdot (\frac{1}{r^{2}}\int rT(r)dr+T(r))$$

where *σ_r_* is the radial thermal stress, (MPa), *σ_θ_* is the circumferential thermal stress, (MPa), *C*_1_ and *C*_2_ are integration constants determined by boundary conditions, *v* is Poisson’s ratio, and *T*(*r*) is the radial temperature distribution function.

#### 2.1.3. Plastic Strain and Material Damage Model

Under repeated high-temperature thermal shocks, plastic deformation occurs inside the material.

The equivalent plastic strain can be expressed as:

(6) $${\bar{\varepsilon }}_{p}=\frac{1}{\sqrt{2(1+\upsilon )}}\sqrt{(\varepsilon_{1}^{p}-\varepsilon_{2}^{p}{)}^{2}+(\varepsilon_{2}^{p}-\varepsilon_{3}^{p}{)}^{2}+(\varepsilon_{3}^{p}-\varepsilon_{1}^{p}{)}^{2}}$$

where ${\bar{\varepsilon }}_{p}$ is the equivalent plastic strain; $\varepsilon_{1}^{p}$, $\varepsilon_{2}^{p}$ and $\varepsilon_{3}^{p}$ are the plastic strain components in the three principal directions, respectively; v is Poisson’s ratio of the material.

In this study, the reported damage penetration depth was identified from the PEEQ distribution. It represents the thickness of the region with significant accumulated plastic deformation rather than an experimentally measured crack propagation depth.

The elastoplastic response was described using a temperature-dependent plastic constitutive relationship implemented in the finite element model. The present study focuses on comparative PEEQ evolution under different thermal-cycle conditions, and the detailed cyclic hardening calibration was beyond the scope of the current work.

For the present comparative analysis, fatigue damage accumulation was evaluated using a linear cumulative damage formulation:

(7) $$D=\sum \frac{n_{i}}{N_{i}}$$

where *D* is the total damage accumulation. *n_i_* is the number of cycles experienced under the *i*-th stress level, and *N_i_* is the fatigue life corresponding to the *i*-th stress level.

The linear cumulative damage formulation was adopted as a first-order comparative framework to evaluate the relative influence of different thermal-cycle parameters rather than to reproduce all nonlinear damage mechanisms occurring under actual service conditions. Accordingly, nonlinear load-sequence effects, creep–fatigue interaction, environmental degradation, and multiaxial damage coupling are not explicitly included in the present damage formulation. Therefore, the calculated damage and fatigue-life results should primarily be interpreted in terms of relative trends among the investigated thermal-cycle conditions rather than as absolute service-life predictions.

The cyclic response in the present study is evaluated in terms of the progressive accumulation of PEEQ with increasing thermal cycles. Accordingly, the discussion uses PEEQ accumulation to describe the simulated plastic-deformation response and does not infer experimentally verified cyclic stabilization or ratcheting behavior.

#### 2.1.4. Comparative Fatigue-Life Assessment Model

Frequent thermal shock loading belongs to low-cycle fatigue behavior.

The Coffin–Manson relationship was adopted as a comparative fatigue-life assessment method to evaluate the relative influence of thermal-cycle parameters on fatigue degradation [23]. Since direct low-cycle fatigue calibration under the identical thermal-cycle history, material state, and mechanical boundary conditions was not available, the calculated fatigue-life values are used to compare the variation trend among different thermal-shock conditions rather than to provide absolute service-life prediction.

(8) Nf=(12)(Δεp2εf′)1c

where *N_f_* is the theoretical predicted life of the material, $\varepsilon_{f}^{\prime }$ is the fatigue ductility coefficient, $\Delta \varepsilon_{p}$ is the plastic strain amplitude, c is the fatigue ductility exponent.

In the present study, the plastic strain amplitude was obtained from the cyclic variation in equivalent plastic strain (PEEQ) during the thermal-shock process. The maximum and minimum plastic strain values within one thermal cycle were extracted from the finite element results, and the half-range value was used as the representative plastic strain amplitude for comparative fatigue-life evaluation:

(9) $$\frac{\Delta \varepsilon_{p}}{2}=\frac{\varepsilon_{p,\max}-\varepsilon_{p,\min}}{2}$$

where $\varepsilon_{p,\max}$ and $\varepsilon_{p,\min}$ represent the maximum and minimum plastic strain values during one thermal-shock cycle, respectively.

Equation (8) is used here to obtain comparative theoretical fatigue-life estimates from the calculated plastic-strain response. Because direct low-cycle-fatigue calibration under the same thermal-cycle history and mechanical boundary conditions was not available, the resulting life values are interpreted as model-based comparative estimates rather than experimentally validated service lives.

### 2.2. Physical Model and Material Parameters

**Figure 1:**
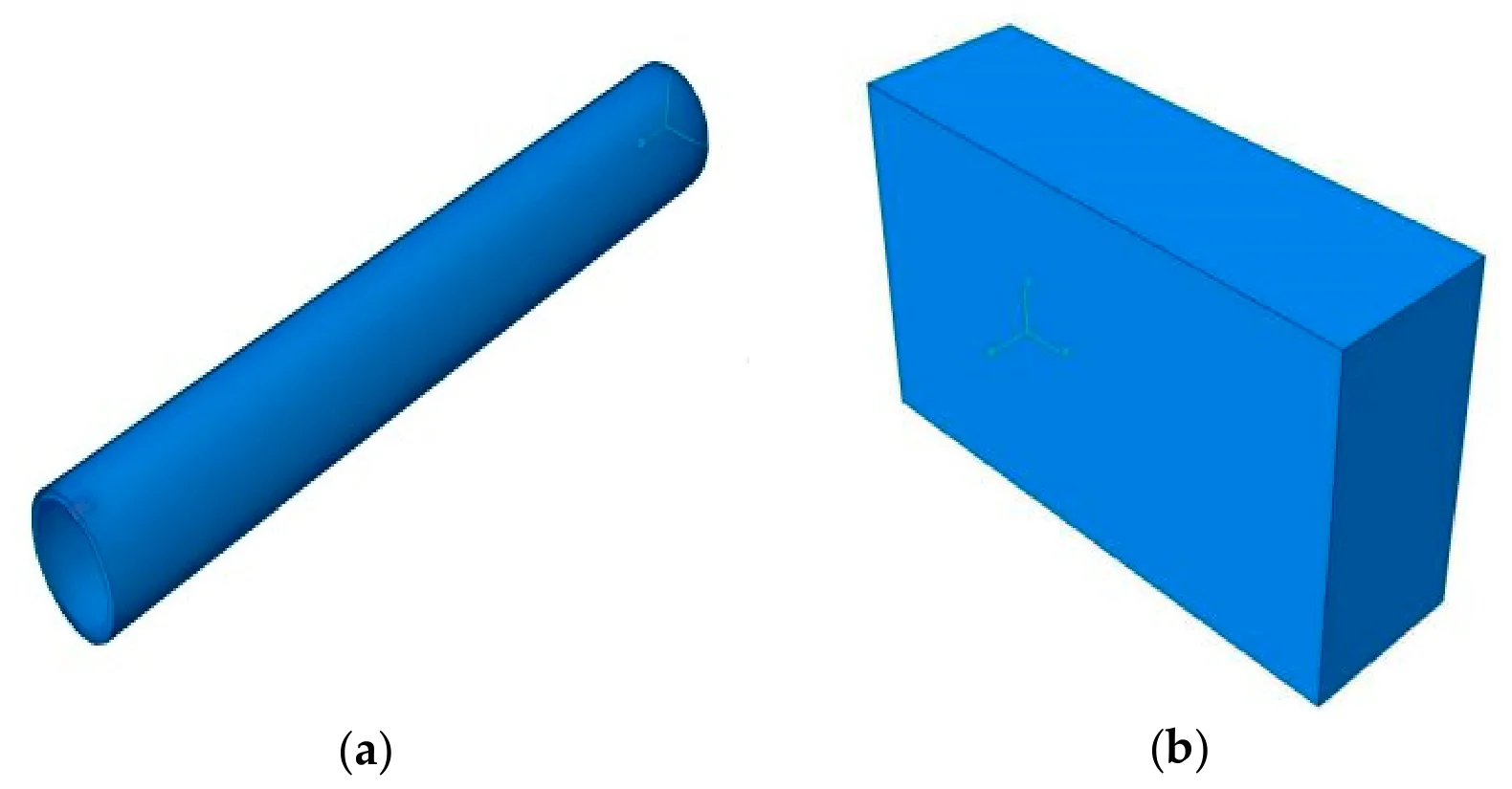
Schematic illustration of the boiler tube and extracted representative tube-wall model. (**a**) Schematic diagram of boiler tube and thermal shock-affected region; (**b**) extracted representative tube-wall model.

During repeated thermal shock loading, the temperature gradient and thermo-mechanical damage of heat-transfer tubes are mainly concentrated within the near-surface region of the tube wall. Since the objective of this study is to investigate the local damage evolution mechanism induced by thermal shock rather than the overall structural deformation of the entire boiler tube, a representative local tube-wall model was extracted for numerical analysis. The boiler tube and the extracted representative tube-wall model are illustrated in Figure 1.

The extracted model represents a small section of the tube wall subjected to cyclic thermal loading. The thickness direction of the model corresponds to the radial direction of the tube wall, which enables the investigation of temperature distribution, thermal stress evolution, and equivalent plastic strain (PEEQ) accumulation along the wall thickness direction.

Therefore, the simplified rectangular geometry preserves the essential thermo-mechanical characteristics associated with thermal shock damage while reducing computational complexity. The dimensions of the representative model were 10 mm × 6 mm × 3 mm.

The heat-resistant steel 12Cr1MoVG was selected as the research material. The temperature-dependent material properties used in the present simulation are summarized in Table 1. The original material-property dataset was provided by the project commissioning party and includes density, Young’s modulus, Poisson’s ratio, thermal conductivity, and specific heat capacity at the available temperature points.

In addition to the thermal properties listed in Table 1, temperature-dependent elastoplastic behavior was considered in the thermo-mechanical simulation. The coefficient of thermal expansion, yield stress, and plastic stress–strain relationship were introduced as temperature-dependent material parameters for calculating the equivalent plastic strain (PEEQ). For temperature points without directly available material data, the corresponding values were obtained by linear interpolation between adjacent temperature levels. Therefore, the material parameters at 700 °C and 750 °C used in the simulation were generated by interpolation to maintain continuous temperature-dependent material behavior.

### 2.3. Boundary Conditions

**Figure 2:**
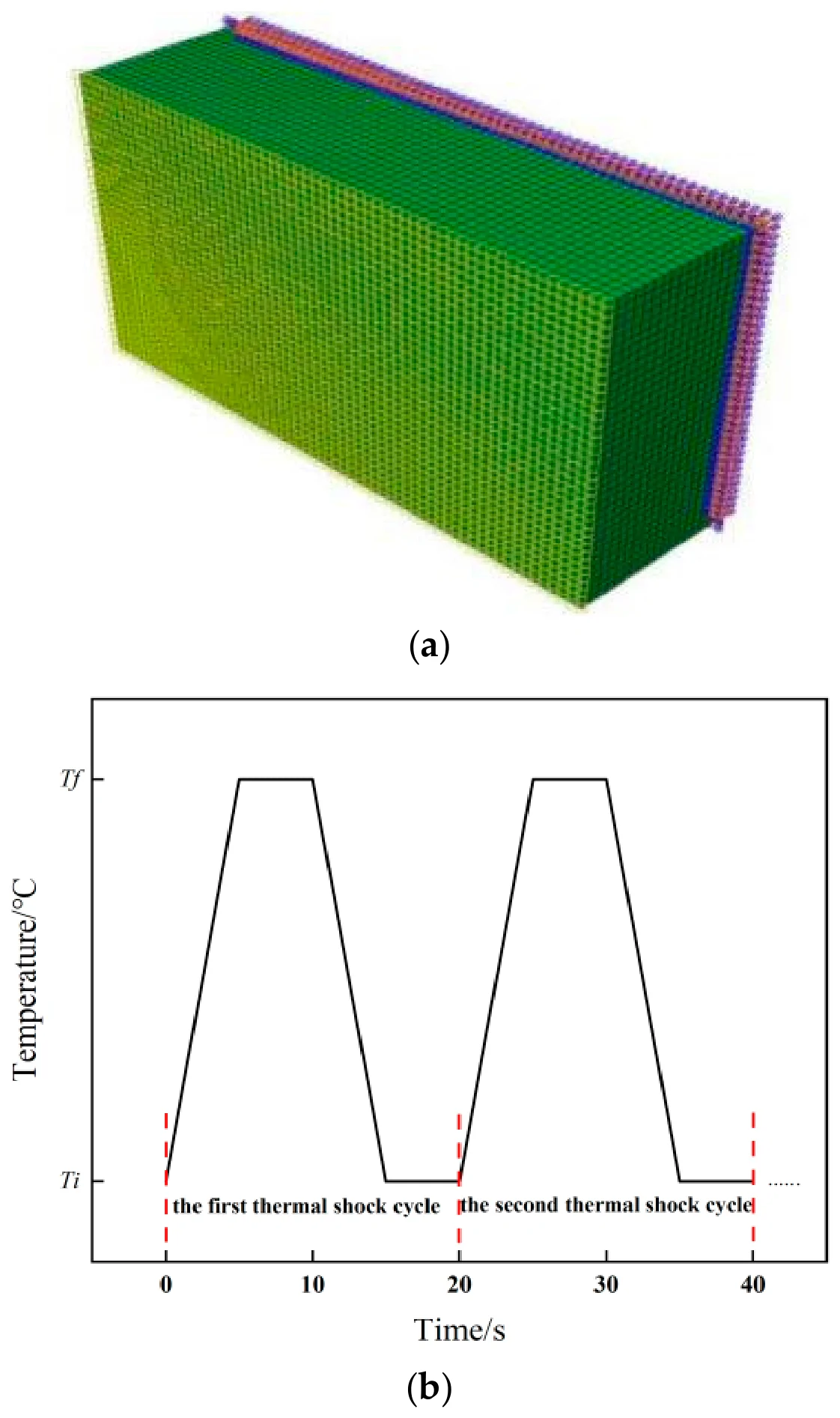
Boundary conditions and thermal-shock loading process: (**a**) mechanical boundary configuration of the representative tube-wall model; (**b**) prescribed thermal-shock temperature history. The red dashed vertical lines indicate the boundaries of individual thermal-shock cycles, and the black dotted line indicates the continuation of cyclic loading.

During the simulation, one surface of the representative tube-wall model was defined as the thermal-shock-exposed surface, on which the prescribed cyclic temperature history was applied. The opposite region was mechanically constrained to represent the restraint provided by the surrounding tube structure and to suppress rigid-body motion. This constraint was introduced as a modeling simplification and does not represent a completely rigid inner surface. Internal pressure was not included in the present model. The mechanical boundary conditions and the prescribed thermal-shock loading process are illustrated in Figure 2.

The representative model is intended to resolve the local through-thickness thermo-mechanical response rather than the global deformation of a complete boiler tube. Consequently, the mechanical restraint is used to provide a consistent local constraint for comparison among thermal-cycle cases. Tube curvature, unrestricted radial expansion, and the multiaxial stress state caused by internal pressure are not reproduced by the present local model.

The transient temperature history was prescribed directly on the thermal-shock-exposed surface without explicitly modeling fluid-solid convective heat transfer. The thermal shock duration and low-temperature holding duration were each set to 5, 10, and 15 s for parametric comparison. All stages of the thermal cycle were treated as transient heat-transfer processes, and a steady-state temperature field was not assumed during the holding stages. The lower temperature of 450 °C represents the low-temperature stage of the cycle, whereas 650–800 °C was used to investigate severe thermal-shock conditions. Creep was not explicitly included in the present model; therefore, the high-temperature results are interpreted primarily as short-duration thermo-mechanical fatigue trends. Temperatures above 850 °C were not considered because additional degradation mechanisms may become significant under such extreme overheating conditions.

For the parametric comparison, the high-temperature thermal-shock duration and the low-temperature holding duration were varied independently among 5, 10, and 15 s, while the remaining model assumptions and boundary definitions were kept unchanged. The imposed surface-temperature history was treated as a transient prescribed-temperature boundary throughout the cycle; no steady-state temperature field was imposed during the holding stages.

### 2.4. Grid Independence Verification and Basic Assumptions

Three-dimensional eight-node thermal-coupled reduced-integration elements (C3D8T) were used for mesh generation in the finite element simulation. Three mesh densities were selected: 31,895 elements; 50,274 elements; 93,646 elements. The influence of mesh density on PEEQ accumulation results under the temperature range of 450–750 °C was investigated. The results showed that when the number of elements exceeded 50,000, the accumulated PEEQ values remained almost unchanged. Therefore, a mesh size of 50,274 elements was selected for subsequent calculations. After verification, the minimum element quality of the model was 0.1021. The average element quality was 0.1102. The total number of mesh elements was 50,274. The mesh independence verification results are shown in Table 2.

During repeated thermal shock loading under different operating conditions, multiple physical processes occur inside the material, including heat transfer, deformation, and damage evolution.

Therefore, several assumptions are introduced [24,25,26,27]:

(1) The material is assumed to be homogeneous and isotropic. The influence of microscopic differences, such as grain orientation, on macroscopic mechanical behavior is neglected.
(2) Plastic deformation is assumed to be completely irreversible. Recovery and recrystallization effects on plastic strain relaxation are not considered.
(3) The local model assumption is adopted because the present study focuses on the thickness-direction damage evolution caused by thermal shock rather than the global deformation behavior of the entire tube.
(4) The mechanical constraint of the inner region is simplified to represent structural restriction and eliminate rigid body motion. The local tube-wall deformation behavior under thermal shock loading is investigated under this simplified constraint condition.
(5) The nonlinear effects caused by different loading sequences and load interactions are neglected.

### 2.5. Model Verification and Literature-Based Comparison

The numerical model was first assessed through the mesh-independence study described in Section 2.4, which confirmed that the predicted PEEQ accumulation was insensitive to further mesh refinement beyond approximately 50,000 elements. Direct thermal-fatigue experiments under exactly the same geometry, thermal-cycle history, and boundary conditions as those used in the present simulations were not available; therefore, one-to-one experimental validation could not be performed.

The relative variation in PEEQ among the three mesh densities is below 0.01%, indicating negligible discretization sensitivity over the investigated mesh range. This provides a quantitative check of numerical mesh convergence, but it does not constitute physical validation of the thermo-mechanical model.

As an alternative qualitative benchmark, the predicted thermo-mechanical response was compared with previously reported thermal-fatigue observations for high-temperature pressure-bearing components. Previous studies have shown that repeated thermal cycling can produce thermal-stress accumulation, near-surface plastic deformation, progressive crack development, and thermal-fatigue degradation [7,8]. These reported trends are qualitatively consistent with the present numerical results, in which PEEQ accumulates progressively under repeated thermal shocks and the predicted damaged region extends with increasing thermal-shock duration.

Therefore, the present verification should be interpreted as numerical consistency and qualitative literature-based benchmarking rather than direct experimental validation. Quantitative validation of the absolute stress, PEEQ, damage depth, and fatigue-life predictions will require dedicated thermal-fatigue or low-cycle-fatigue experiments conducted under equivalent thermal and mechanical boundary conditions.

Accordingly, the absolute values of thermal stress, PEEQ, damage penetration depth, and theoretical fatigue life should be regarded as model-dependent estimates. Their quantitative uncertainty is associated with the simplified geometry and boundary conditions, the limited elevated-temperature material-property data, the omission of internal pressure and explicit convection, and the absence of direct experimental calibration under equivalent thermal-cycle conditions.

## 3. Results and Analysis

By changing the thermal shock duration and low-temperature holding duration within each thermal shock cycle, numerical simulations were performed to investigate the fatigue behavior of boiler heat-resistant materials under different cyclic loading conditions. The evolution characteristics of thermal stress, PEEQ accumulation at different depths, failure behavior, and theoretical life variation in the heat-resistant steel were analyzed. The fatigue damage accumulation mechanism of the material was further evaluated.

### 3.1. Effect of Thermal Shock Duration on Material Fatigue Damage

**Figure 3:**
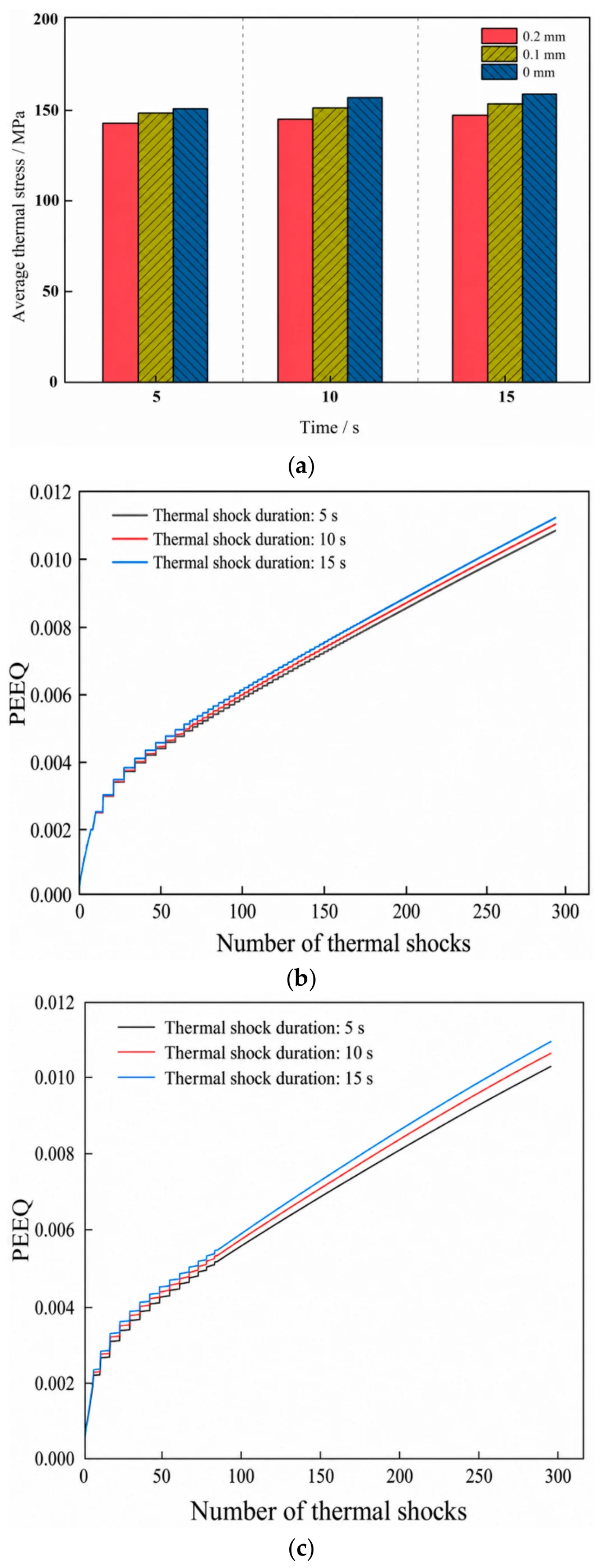

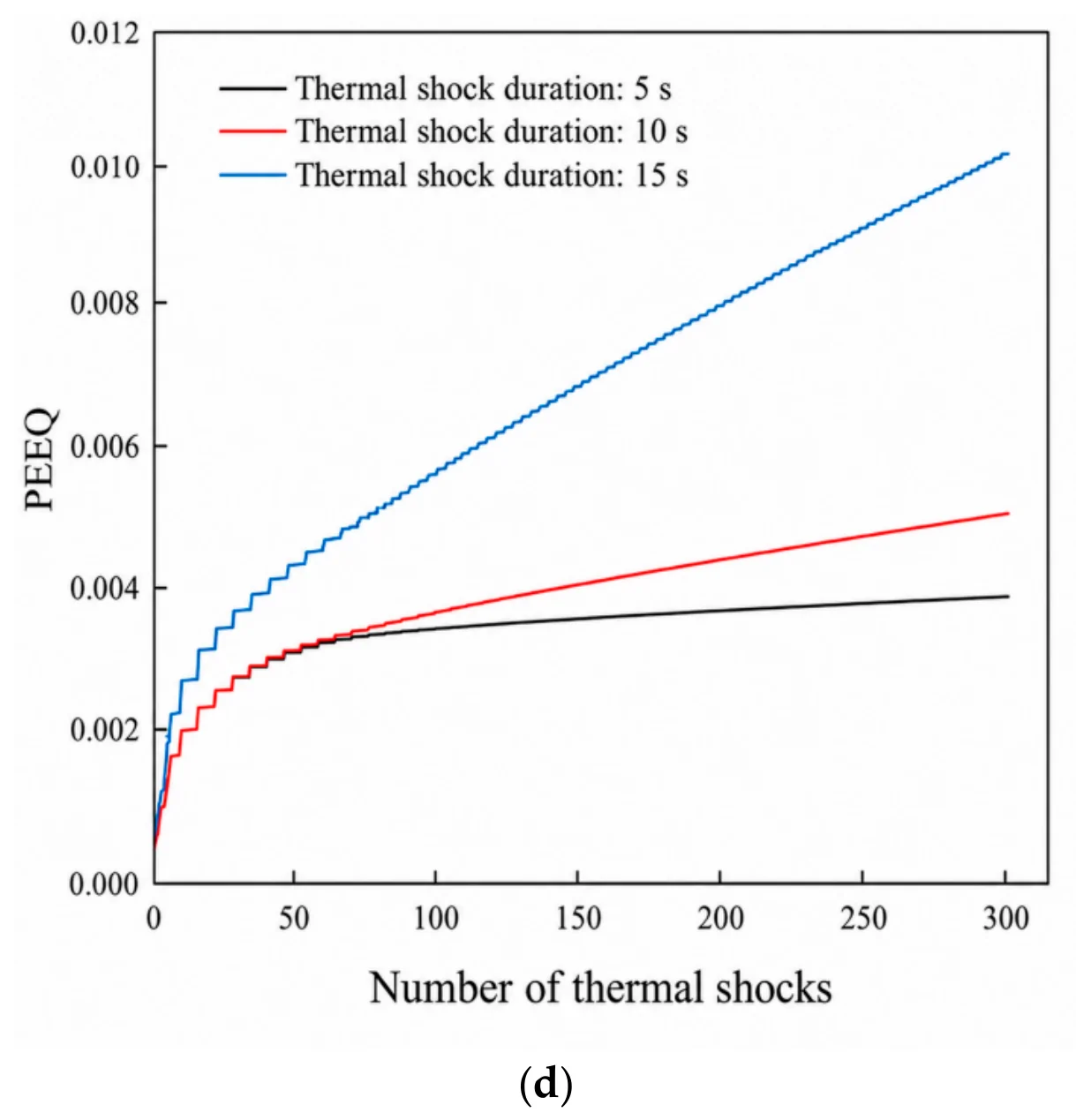
Effect of high-temperature thermal-shock duration on the thermo-mechanical response of 12Cr1MoVG steel: (**a**) thermal stress; (**b**) PEEQ under different thermal shock durations at the thermal-shock-exposed surface; (**c**) PEEQ under different thermal shock durations at 1 mm below the thermal-shock-exposed surface; (**d**) PEEQ under different thermal shock durations at 2.5 mm below the thermal-shock-exposed surface.

**Figure 4:**
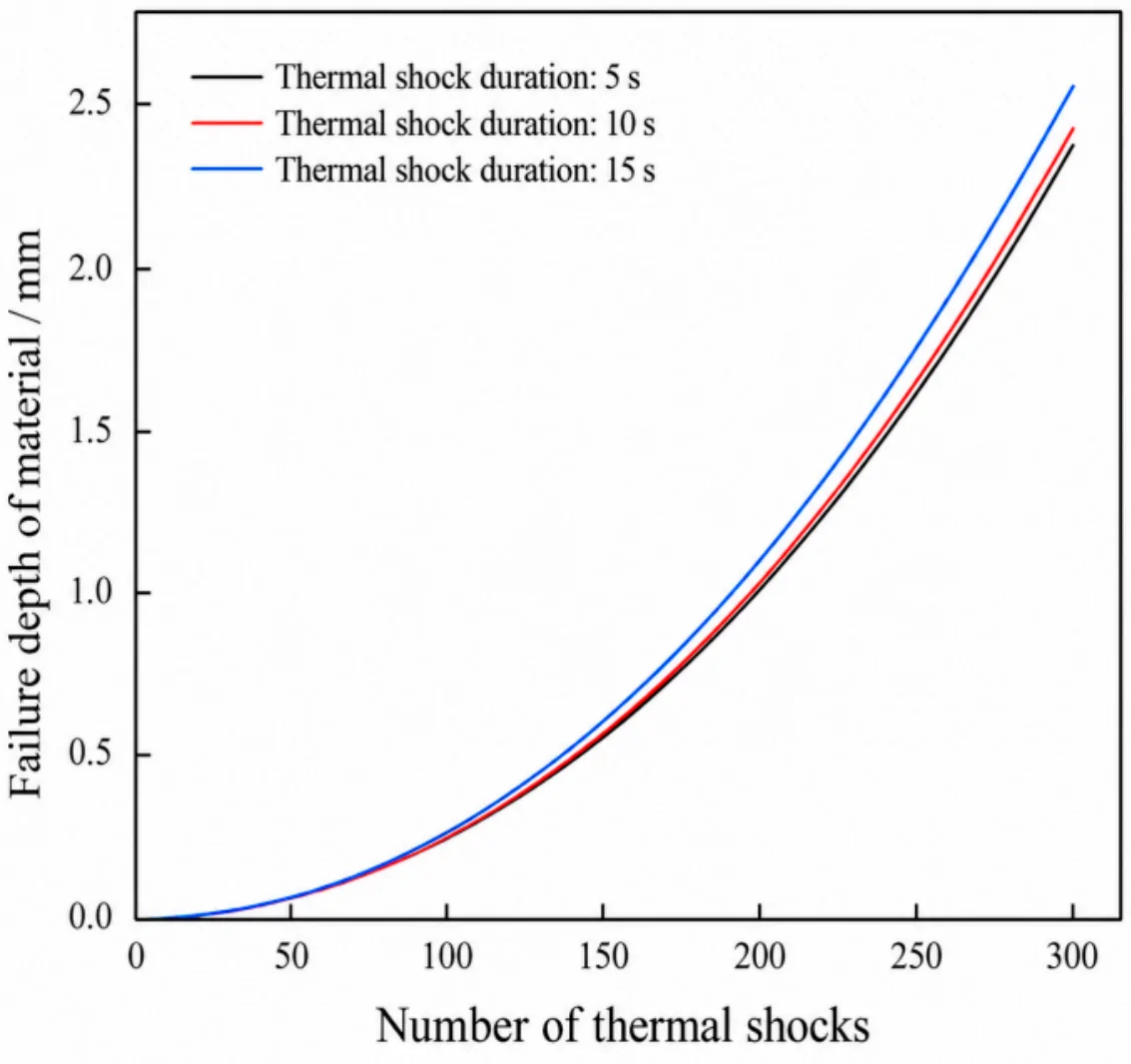
Damage penetration depth under different high-temperature thermal-shock durations.

Equivalent plastic strain (PEEQ) is defined based on the Mises yield criterion. It converts complex strains under three-dimensional stress states into equivalent strains generated by uniaxial tension, and PEEQ is adopted to comprehensively characterize the overall deformation degree of materials. The PEEQ distribution cloud can identify the dominant plastic deformation zones and failure morphologies at nodes. Figure 3 illustrates the variation laws of calculated thermal stress and PEEQ for 12Cr1MoVG heat-resistant steel under different thermal shock durations, while Figure 4 presents the change in material damage penetration depth of the steel with varying thermal shock durations. As the high-temperature thermal shock duration increases, the damage penetration depth of the material along the thickness direction rises significantly from 2.24 mm to 2.60 mm. Meanwhile, the PEEQ accumulation rate at different material depths also varies remarkably with prolonged thermal shock loading. This phenomenon mainly arises because extended high-temperature thermal shock accelerates heat diffusion and deepens the heat penetration along the thickness direction, which raises the average thermal stress at all depths of the material. This indicates that thermal shock duration does not simply increase the magnitude of thermal stress but also changes the spatial distribution and accumulation characteristics of plastic deformation, thereby controlling the evolution of the damaged region. The thermal stress at the material surface increases from 151.21 MPa to 158.35 MPa; at a depth of 1 mm beneath the surface, thermal stress rises from 148.35 MPa to 153.16 MPa; at a depth of 2.5 mm below the surface, thermal stress grows from 142.65 MPa to 146.76 MPa. The increase in thermal stress enhances the tendency for local plastic deformation and consequently promotes PEEQ accumulation. During the subsequent cooling stage, thermal contraction changes the local stress state and further contributes to cycle-by-cycle plastic-strain accumulation. Nevertheless, deep material regions are not directly subjected to thermal shock and only form a secondary temperature field. Compared with the thermal-shock-exposed surface and the adjacent subsurface region, material located farther from the exposed surface exhibits a lower PEEQ accumulation rate and less severe damage. At a depth of 2.5 mm below the thermal-shock-exposed surface, the PEEQ accumulation rate of 12Cr1MoVG steel remains relatively low during thermal shock loading, indicating that plastic-strain accumulation is considerably weaker at this depth. However, as the thermal shock duration increases, the PEEQ accumulation rate at 2.5 mm gradually increases, demonstrating that prolonged thermal shock promotes deeper damage penetration along the material thickness direction.

### 3.2. Effect of Low-Temperature Holding Duration on Material Fatigue Damage

**Figure 5:**
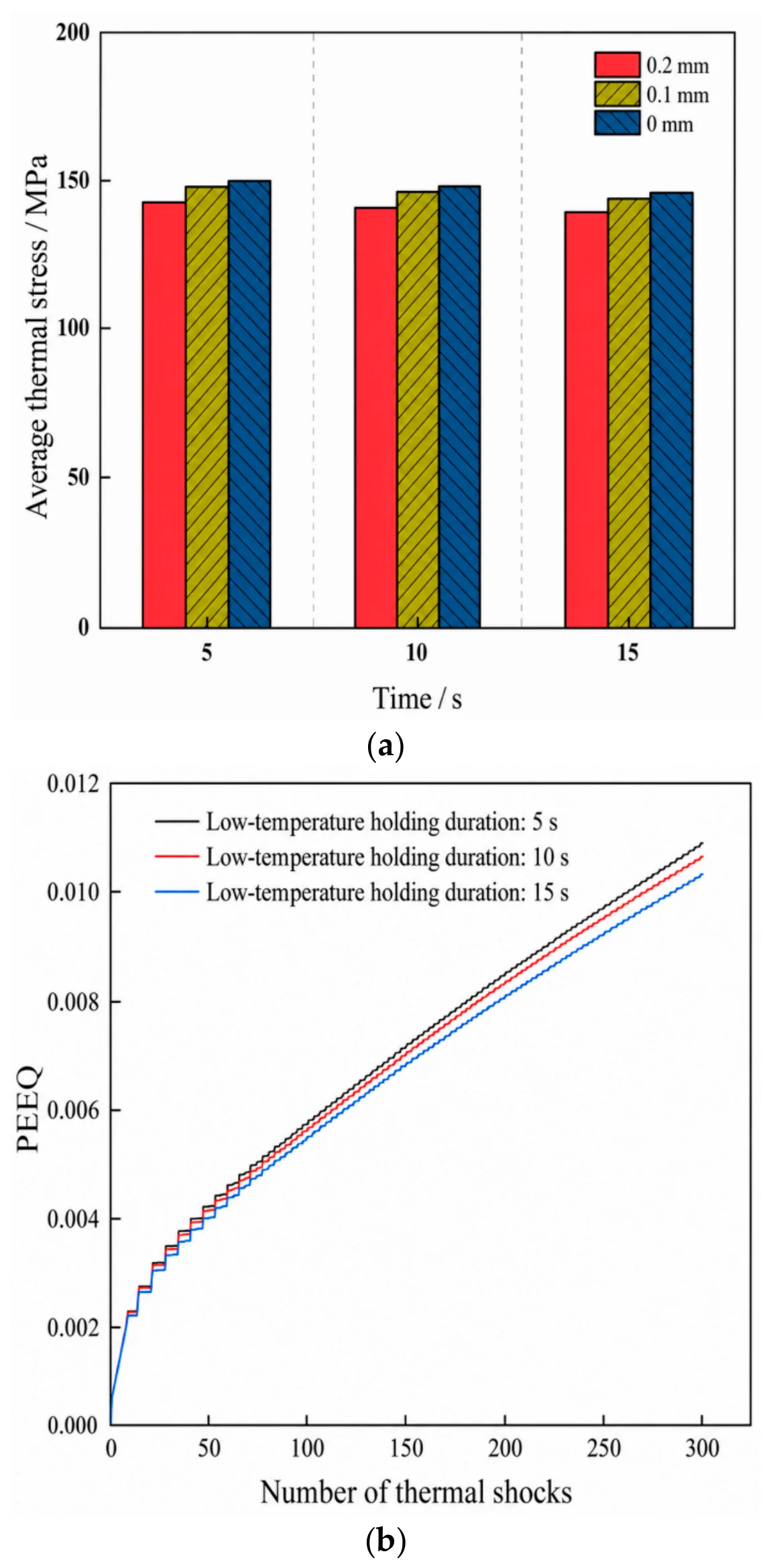

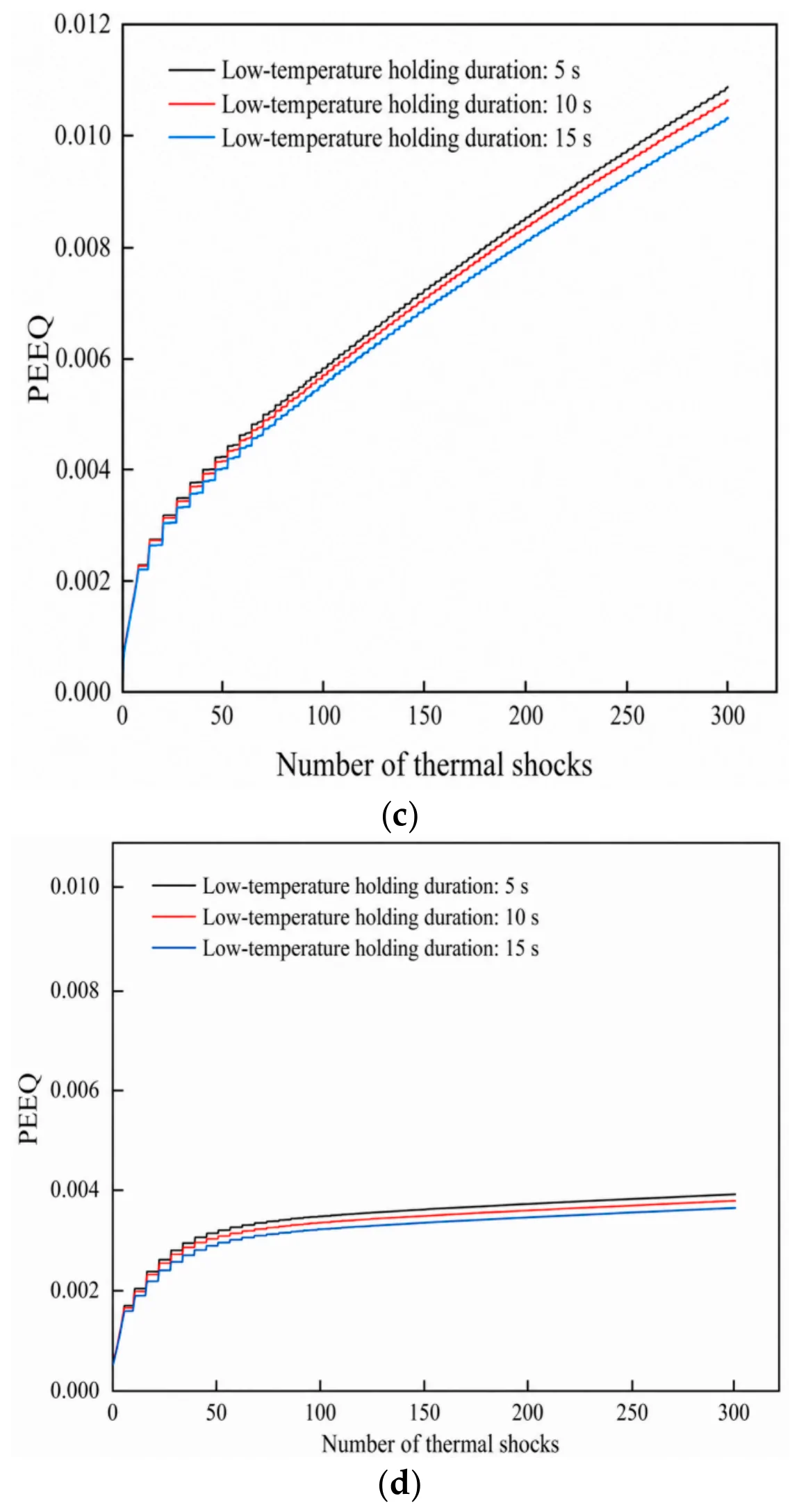
Effect of low-temperature holding duration on the thermo-mechanical response of 12Cr1MoVG steel: (**a**) thermal stress; (**b**) PEEQ under different low-temperature holding durations at the thermal-shock-exposed surface; (**c**) PEEQ under different low-temperature holding durations at 1 mm below the thermal-shock-exposed surface; (**d**) PEEQ under different low-temperature holding durations at 2.5 mm below the thermal-shock-exposed surface.

**Figure 6:**
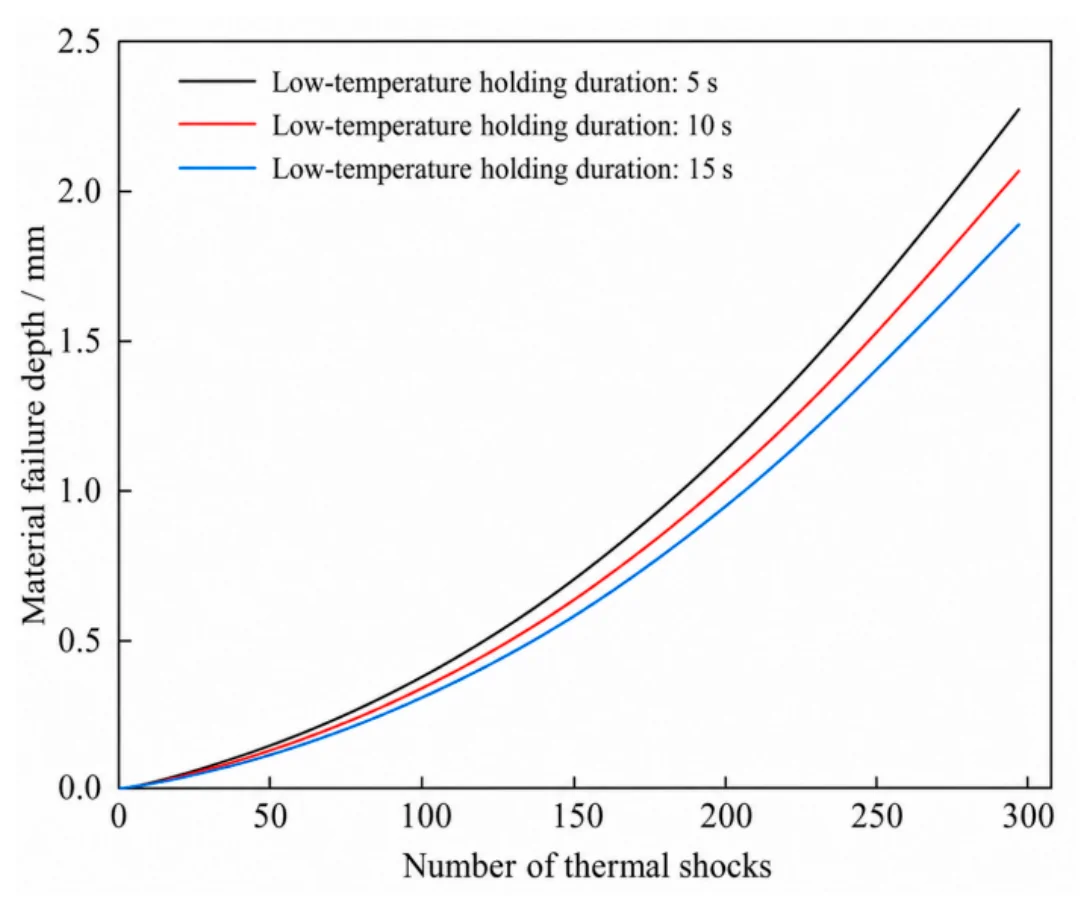
Material failure depth.

As shown in Figure 5, the period during which the material is maintained at 450 °C is defined as the low-temperature holding period. When the low-temperature holding duration increases from 5 s to 15 s, the PEEQ accumulation rate gradually decreases at different depths of the material. This behavior is mainly attributed to the decrease in thermal stress induced by the prolonged low-temperature holding duration. Specifically, the thermal stress decreases from 150.24 MPa to 146.48 MPa at the material surface, from 148.36 MPa to 144.48 MPa at a depth of 1 mm below the surface, and from 143.24 MPa to 139.85 MPa at a depth of 2.5 mm below the surface. The reduction in thermal stress at different depths makes it more difficult for the material to undergo significant plastic deformation. Therefore, the tendency for additional plastic deformation is reduced, resulting in a lower PEEQ accumulation rate as the low-temperature holding duration increases. Consequently, the PEEQ accumulation rate continuously decreases with increasing low-temperature holding duration. It should be noted that the PEEQ growth rate at 2.5 mm below the surface is extremely low and gradually approaches a saturation value. This indicates that fatigue damage is relatively limited in this region under the investigated conditions. As shown in Figure 6, the damage penetration depth gradually decreases from 2.24 mm to 2.03 mm, indicating a continuous reduction in the thickness of the damaged layer. Compared with the effect of extending the thermal shock duration, the variation in damage penetration depth induced by increasing the low-temperature holding duration is relatively limited. This is mainly because, although the low-temperature holding duration increases, the temperature remains as high as 450 °C. The reduced temperature difference between the material and its surroundings decreases the cooling rate and results in only a slight variation in thermal stress amplitude. Therefore, the reduction in failure depth remains relatively small.

### 3.3. Life Prediction of the Material

**Figure 7:**
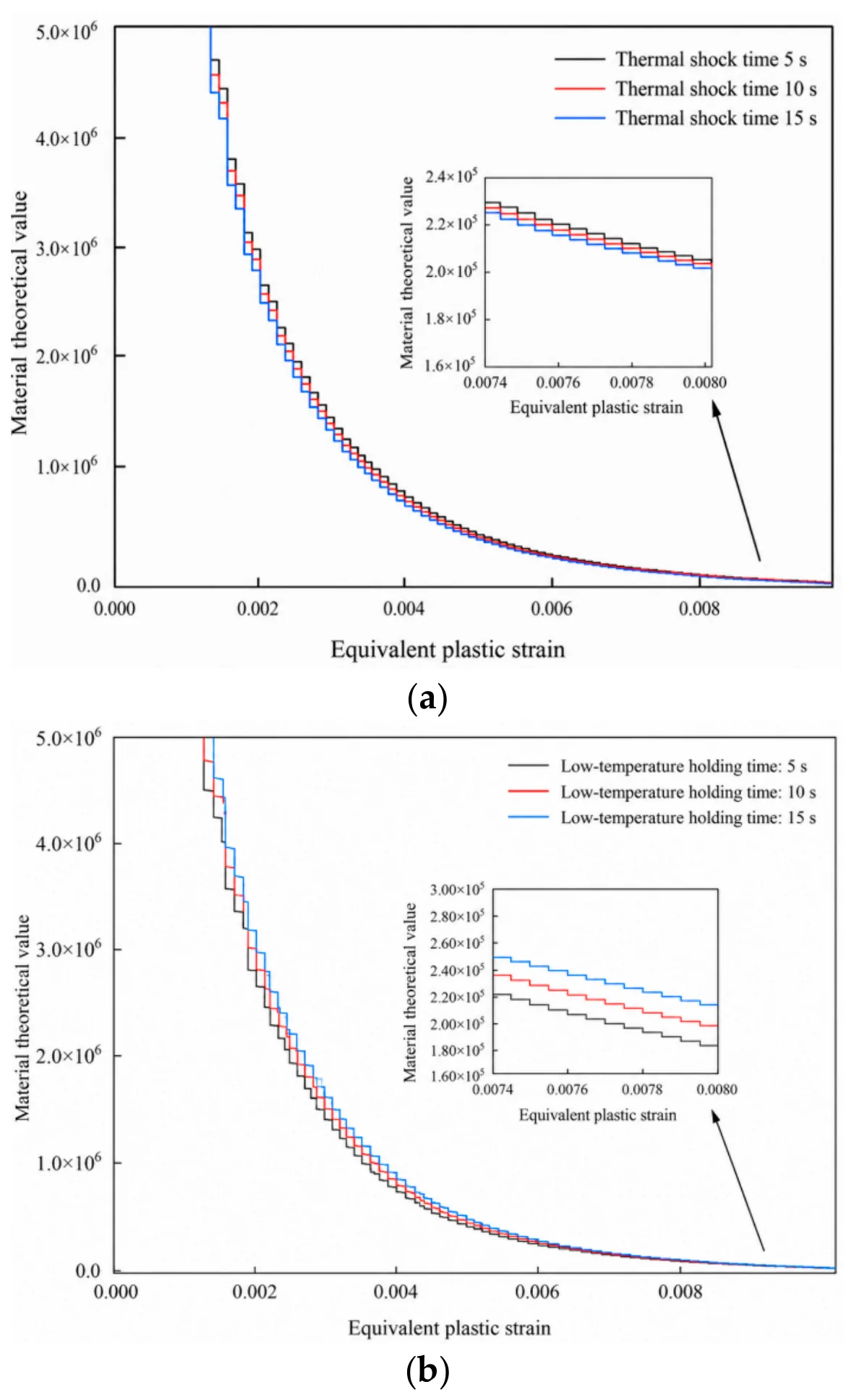
The influence of different cycle durations on the theoretical lifespan of the material. (**a**) The impact of the duration of thermal shock on the theoretical lifespan of the material; (**b**) the influence of the duration of low-temperature exposure on the theoretical lifespan of the material.

The theoretical fatigue-life estimates of the material under different thermal-shock cycle conditions are shown in Figure 7. When the high-temperature thermal shock duration within each cycle increases from 5 s to 15 s, the reduction rate of the predicted theoretical life becomes faster. The theoretical life decreases from 1.82 × 10^5^ cycles to 1.72 × 10^5^ cycles. The decline of theoretical life is mainly attributed to the continuous expansion of the material during prolonged high-temperature thermal shock. Because the material remains in a sustained expansion state, the elastic strain cannot be effectively recovered within a short period. Consequently, PEEQ accumulation continuously increases. The accelerated accumulation of material damage leads to a gradual reduction in the predicted theoretical life. When the low-temperature holding duration increases from 5 s to 15 s, the theoretical life of the material increases from 1.81 × 10^5^ cycles to 1.94 × 10^5^ cycles. The increase in theoretical life is mainly caused by the gradual reduction in temperature gradient along the thickness direction during the extended low-temperature holding period. The predicted damage-evolution trend is qualitatively consistent with previously reported thermal-fatigue behavior of high-temperature pressure-bearing components, in which repeated temperature fluctuations promote thermal-stress accumulation, crack development, and progressive fatigue degradation [7,8]. Within the investigated parameter range, the numerical results suggest that prolonged high-temperature thermal-shock exposure should be avoided where possible, whereas an appropriate increase in the low-temperature holding duration may help reduce thermal-stress amplitude, suppress plastic-strain accumulation, and delay fatigue damage development.

### 3.4. Limitations and Future Perspectives

Although the present numerical model provides insight into thermal-shock-induced damage evolution of 12Cr1MoVG heat-resistant steel, several limitations should be acknowledged. First, the 10 mm × 6 mm × 3 mm model represents a local tube-wall segment rather than the complete tubular component. Consequently, tube curvature, global structural deformation, and the complete radial expansion behavior of the tube are not explicitly reproduced. The mechanical constraint applied to the opposite region was used to represent the restraint of the surrounding structure and to stabilize the local model, but it cannot fully reproduce the mechanical compliance of an actual tube under service conditions. In addition, internal pressure was not included as a mechanical load. Therefore, the calculated stress and PEEQ distributions do not represent the complete multiaxial stress state occurring in an operating pressurized boiler tube. The omission of internal pressure may affect the magnitude and multiaxiality of the predicted stress–strain response and consequently introduces uncertainty into the absolute fatigue-life prediction. Accordingly, the present results are primarily used to compare the relative influence of thermal shock duration and low-temperature holding duration under otherwise identical numerical conditions.

In addition, the fatigue-damage formulation adopted in Equation (7) is based on linear cumulative damage and does not explicitly account for nonlinear load-sequence-dependent damage evolution. Consequently, the predicted fatigue lives are intended mainly for comparative evaluation of the investigated thermal-cycle parameters. Future work should incorporate nonlinear damage formulations and creep–fatigue interaction models to improve quantitative life prediction under service conditions.

Second, the present model does not consider creep damage and environmental degradation effects, which may become important during long-term high-temperature service. In addition, the material properties adopted in the simulation are limited to the available temperature range, and further experimental measurements are required to reduce uncertainty at elevated temperatures. In addition, explicit fluid-solid convective heat transfer, circumferentially non-uniform thermal loading, and axial load variations were not included in the present representative model.

Finally, direct experimental validation under thermal-cycle conditions equivalent to those used in the present simulations was not available. Although the numerical trends were qualitatively benchmarked against published thermal-fatigue observations, the absolute values of stress, PEEQ, damage depth, and fatigue life remain to be quantitatively validated. Future work will therefore include dedicated thermal-fatigue and low-cycle-fatigue experiments together with more comprehensive thermo-mechanical damage models incorporating internal pressure and creep-fatigue interaction.

The reported damage-penetration depths and fatigue-life values should therefore be interpreted as comparative numerical indicators within the adopted modeling framework rather than as experimentally calibrated failure thresholds or absolute service-life predictions. Independent reproduction and quantitative validation will require a fully documented temperature-dependent elastoplastic constitutive dataset, an explicitly reported cyclic hardening formulation, calibrated fatigue parameters, and a clearly defined damage-depth criterion.

## 4. Conclusions

The damage evolution behavior and theoretical life characteristics of 12Cr1MoVG heat-resistant steel under different thermal shock cycle conditions were investigated through finite element simulations.

The main conclusions are summarized as follows:

(1) Increasing the thermal shock duration causes the damage penetration depth of the material to increase from 2.24 mm to 2.60 mm. The accumulation rate of PEEQ increases with increasing thermal shock duration. Therefore, the fatigue damage of the material is accelerated.
(2) Increasing the low-temperature holding duration reduces the damage penetration depth of the material from 2.24 mm to 2.03 mm. The accumulation rate of PEEQ decreases as the low-temperature holding duration increases. Therefore, the fatigue damage evolution of the material is effectively delayed.
(3) When the thermal shock duration increases from 5 s to 15 s, the theoretically predicted life decreases from 1.82 × 10^5^ cycles to 1.72 × 10^5^ cycles. When the low-temperature holding duration increases from 5 s to 15 s, the theoretical predicted life increases from 1.81 × 10^5^ cycles to 1.94 × 10^5^ cycles.

## Figures and Tables

**Table 1:** Parameters of 12Cr1MoVG material.

Temperature °C	Density kg/m^3^	Young’s Modulus E/GPa	Poisson’s Ratio ν	Thermal Conductivity W/(m·°C)	Specific Heat Capacity J/(kg·°C)
20	7860	225	0.29	43	420
100	7840	221	0.29	41	460
300	7790	214	0.3	37	490
450	7740	207	0.3	35	515
500	7670	206.2	0.3	33	530
550	7610	195.3	0.31	31	542
600	7520	181	0.31	28	550
650	7460	143	0.31	26	562
700	-	-	-	-	-
750	-	-	-	-	-

Note: The material-property dataset was supplied by the project commissioning party. Data at 700 °C and 750 °C were not available in the original dataset.

**Table 2:** Grid independence verification.

Case	Number of Elements	PEEQ Accumulation	Difference/%
1	93,646	0.18348	0.0056
2	50,274	0.18347	Standard
3	31,895	0.18346	0.0052

## Data Availability

The original contributions presented in this study are included in the article. Further inquiries can be directed to the corresponding author.
